# The role of multidetector CT angiography and 3D postprocessing imaging in the diagnosis and investigation of bronchopulmonary sequestration

**DOI:** 10.1002/ccr3.1394

**Published:** 2018-01-31

**Authors:** Eleftherios Spartalis, Michael Spartalis, Demetrios Moris, Antonios Athanasiou, Theodore Troupis, Periklis Tomos

**Affiliations:** ^1^ Laboratory of Experimental Surgery and Surgical Research University of Athens Medical School Athens Greece; ^2^ Department of Surgery The Ohio State University Comprehensive Cancer Center The Ohio State University Columbus Ohio USA; ^3^ Department of Surgery Mercy University Hospital Cork Ireland; ^4^ Faculty of Medicine Department of Anatomy National and Kapodistrian University of Athens Athens Greece; ^5^ Department of Thoracic Surgery “Attikon” Hospital Athens Medical School Athens Greece

**Keywords:** Bronchopulmonary sequestration, computed tomography

## Abstract

Congenital bronchopulmonary malformations are usually asymptomatic. Precise multimodality imaging plays an essential role in the identification of rare cardiothoracic entities, offering excellent imaging quality and the decisive diagnosis.

## Case Presentation

A 68‐year‐old male patient presented to our department complaining of eight‐month‐lasting dry cough. Physical examination showed nothing of significance. Chest X‐ray demonstrated lobar atelectasis of the left lung. Chest CT scan confirmed the initial diagnosis, and per os antimicrobial medication was prescribed.

**Figure 1:**
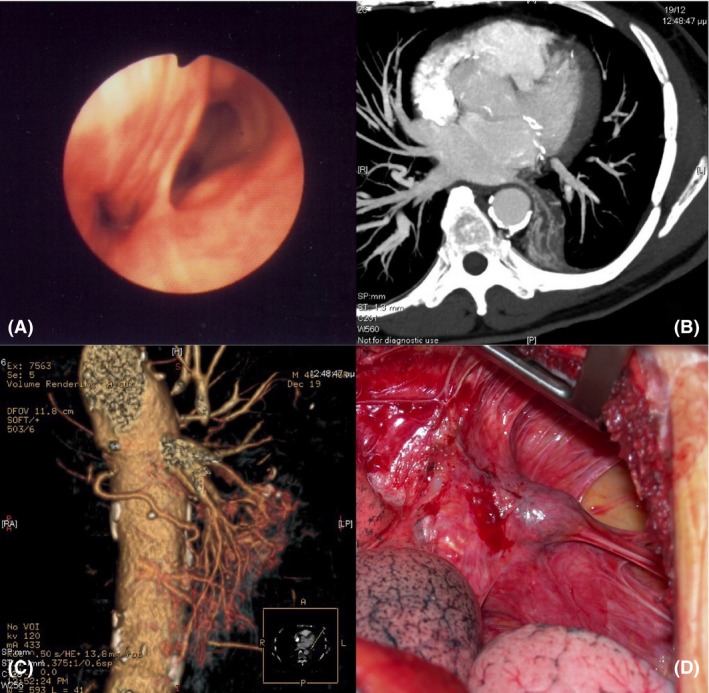
(A) Bronchoscopy reveals an ectopic port of the left main bronchus. (B) Chest computed tomography image shows ectopic perfusion of the lesion from the aorta. (C) Three‐dimensional maximum intensity projection computed tomography image shows ectopic perfusion of the lesion from the aorta. (D) The lesion during left posterolateral thoracotomy.

After 8 months, and due to persistent symptoms, a new CT scan was performed, which showed no changes of the lesion in the left paravertebral space. The patient underwent bronchoscopy which identified an ectopic port of the left main bronchus (Fig. 1A). Maximum intensity projection CT and three‐dimensional postprocessing reconstruction that followed showed ectopic perfusion from the aorta (Fig. 1B and C). The patient underwent resection of the lesion via left posterolateral thoracotomy (Fig. 1D). The histological examination verified the presence of a sequestration, a rare type of bronchopulmonary foregut malformation.

## Authorship

Eleftherios Spartalis: involved in conception and design of the research and writing of the manuscript. Eleftherios Spartalis and Michael Spartalis: involved in acquisition of data. Eleftherios Spartalis, Michael Spartalis, Demetrios Moris and Antonios Athanasiou: analyzed and interpreted the data. Theodore Troupis and Periklis Tomos: performed critical revision of the manuscript for intellectual content.

## Conflict of Interest

The authors report no financial relationships or conflict of interests regarding the content herein.

